# Interrupted *versus* continuous suture technique for biliary-enteric anastomosis: randomized clinical trial

**DOI:** 10.1093/bjsopen/zrac163

**Published:** 2023-02-01

**Authors:** Lena Seifert, Janusz von Renesse, Adrian M Seifert, Dorothée Sturm, Ronny Meisterfeld, Nuh N Rahbari, Christoph Kahlert, Marius Distler, Jürgen Weitz, Christoph Reissfelder

**Affiliations:** Department of Visceral, Thoracic and Vascular Surgery, University Hospital Carl Gustav Carus, TU Dresden, Dresden, Germany; National Center for Tumor Diseases (NCT), Partner Site Dresden, Heidelberg, Germany; German Cancer Consortium (DKTK), German Cancer Research Center (DKFZ), Partner Site Dresden, Heidelberg, Germany; Department of Visceral, Thoracic and Vascular Surgery, University Hospital Carl Gustav Carus, TU Dresden, Dresden, Germany; Department of Visceral, Thoracic and Vascular Surgery, University Hospital Carl Gustav Carus, TU Dresden, Dresden, Germany; National Center for Tumor Diseases (NCT), Partner Site Dresden, Heidelberg, Germany; German Cancer Consortium (DKTK), German Cancer Research Center (DKFZ), Partner Site Dresden, Heidelberg, Germany; Department of Surgery, Medical Faculty Mannheim, University Medical Center Mannheim, Heidelberg University, Mannheim, Germany; Department of Visceral, Thoracic and Vascular Surgery, University Hospital Carl Gustav Carus, TU Dresden, Dresden, Germany; Department of Surgery, Medical Faculty Mannheim, University Medical Center Mannheim, Heidelberg University, Mannheim, Germany; Department of Visceral, Thoracic and Vascular Surgery, University Hospital Carl Gustav Carus, TU Dresden, Dresden, Germany; National Center for Tumor Diseases (NCT), Partner Site Dresden, Heidelberg, Germany; German Cancer Consortium (DKTK), German Cancer Research Center (DKFZ), Partner Site Dresden, Heidelberg, Germany; Department of Visceral, Thoracic and Vascular Surgery, University Hospital Carl Gustav Carus, TU Dresden, Dresden, Germany; Department of Visceral, Thoracic and Vascular Surgery, University Hospital Carl Gustav Carus, TU Dresden, Dresden, Germany; National Center for Tumor Diseases (NCT), Partner Site Dresden, Heidelberg, Germany; German Cancer Consortium (DKTK), German Cancer Research Center (DKFZ), Partner Site Dresden, Heidelberg, Germany; Department of Surgery, Medical Faculty Mannheim, University Medical Center Mannheim, Heidelberg University, Mannheim, Germany

## Abstract

**Background:**

Biliary-enteric anastomosis (BEA) can be performed using continuous or interrupted suture techniques, but high-quality evidence regarding superiority of either technique is lacking. The aim of this study was to compare the suture techniques for patients undergoing BEA by evaluating the suture time as well as short- and long-term biliary complications.

**Methods:**

In this single-centre randomized clinical trial, patients scheduled for elective open procedure with a BEA between 21 January 2016 and 20 September 2017 were randomly allocated in a 1:1 ratio to have the BEA performed with continuous suture (CSG) or interrupted suture technique (ISG). The primary outcome was the time required to complete the anastomosis. Secondary outcomes were BEA-associated postoperative complications with and without operative revision of the BEA, including bile leakage, cholestasis, and cholangitis, as well as morbidity and mortality up to day 30 after the intervention and survival.

**Results:**

Altogether, 82 patients were randomized of which 80 patients received the allocated intervention (39 in ISG and 41 in CSG). Suture time was longer in the ISG compared with the CSG (median (interquartile range), 22.4 (15.0–28.0) min *versus* 12.0 (10.0–17.0) min, OR 1.26, 95 per cent c.i. 1.13 to 1.40; unit of increase of 1 min; *P* < 0.001). Short-term and long-term biliary complications were similar between groups. The incidence of bile leakage (6 (14.6 per cent) *versus* 4 (10.3 per cent), *P* = 0.738) was comparable between groups. No anastomotic stenosis occurred in either group.

**Conclusion:**

Continuous suture of BEA is equally safe, but faster compared with interrupted suture.

**Registration number:**

NCT02658643 (http://www.clinicaltrials.gov).

## Teaser

A randomized clinical trial comparing the benefits and risks of continuous and interrupted suture techniques for patients undergoing biliary-enteric anastomosis showed no differences for short-term and long-term biliary complications. Suture time for biliary-enteric anastomosis was significantly reduced in the continuous suture group compared with the interrupted suture group.

## Introduction

A biliary-enteric anastomosis (BEA), such as hepaticojejunostomy or choledochojejunostomy is an anastomosis between the common hepatic duct (CHD) or the common bile duct (CBD) and the upper third of the jejunum^1^. Indications for a BEA include total or partial resection of the pancreas and the duodenum for benign and malignant pancreatic tumours, tumours of the biliary tract or duodenum, and biliary tract reconstructions during liver transplantation or advanced liver resections^2^. Further, a BEA can be required to manage the injury of the biliary tract and infectious and traumatic strictures of the biliary tract^3^. A rare but severe short-term postoperative complication after BEA is bile leakage, which increases the morbidity and duration of hospital stay. A previous study has shown that clinically relevant biliary leakage after a hepaticojejunostomy occurs in 2.3 per cent of patients and necessitated antibiotic treatment and maintenance of perioperatively placed drains in 15 per cent, percutaneous transhepatic cholangial drainage (PTCD) in 69 per cent, and relaparotomy with surgical drainage in 18 per cent of patients^4–6^. Only 38 per cent of the blood supply for the bile duct ran downwards from the CHD (paralleling the bile flow), in some patients this was through an additional retroportal artery, and in only 2 per cent, this came from the hepatic artery itself. Therefore, an increased length of the remaining bile duct and a greater distance to the biliary confluence have a higher risk for disrupted blood supply of the anastomosis and consecutive biliary leakage, depending on the type of surgery^7,8^. Anastomotic stricture is a long-term complication that can cause cholestasis with an increased risk of cholangitis, liver abscess formation, secondary biliary cirrhosis, and liver failure^9–11^. BEA can be performed using a continuous suture (CS) or an interrupted suture (IS) technique depending on the surgeon’s experience and preference, the tissue texture, and anatomical characteristics, such as the diameter of the bile duct^12–15^. The IS technique is preferentially used for small and fragile bile ducts. Both techniques are well established methods, but there are limited data comparing the two techniques with regard to complication rates^10^. A retrospective study of patients receiving a right lobe living-donor liver transplantation showed higher incidence of bile leakage using the IS technique, but a lower rate of biliary strictures^16^. A further study, comparing IS and CS techniques in patients receiving whole liver transplantation showed no difference in postoperative complications^12^. A survey among 102 hospitals in Germany revealed that preferential use of either technique is distributed heterogeneously among surgeons; however, both techniques are considered as part of routine practice^13^. A multivariate analysis identified preoperative radiochemotherapy and preoperative low cholinesterase levels, as well as biliary complications after liver transplantation and simultaneous liver resection, as risk factors for bile leakage after BEA independent of the suture technique^17^. A further study showed no differences between the techniques regarding short-term and long-term outcomes in patients receiving pancreaticoduodenectomy or total pancreatectomy^18^. This study is the first prospective, randomized clinical trial that aimed to evaluate the short-term and long-term benefits and risks of different suture techniques for BEA.

## Methods

### Trial design and randomization

This study was approved by the ethics committee of the Technical University Dresden (ethics approval number EK419092015) and complied with the Declaration of Helsinki. The randomized clinical study was registered before inclusion of the first patient at www.clinicaltrials.gov (NCT02658643) to ensure transparency of the design and outcomes. Reporting of the trial complied with the recommendations of the CONSORT statement^19^. The randomization sequence was defined without stratification factors using block randomization with a computer by trial personnel not involved in treatment or outcome assessment of the participants using R statistics. Treatment allocation was performed using consecutively numbered opaque sealed envelopes that were packed by a person who was not involved in the trial later on. Patients were recruited to the trial in the outpatient clinic by a study doctor. During surgery the diameter of the bile duct and the distance to the biliary confluence were measured with a ruler, before the envelope was opened and the anastomosis was performed. Patients were assigned in a 1:1 ratio to the CS group (CSG) or the IS group (ISG) depending on the technique. The BEA was performed as a hepaticojejunostomy in all patients. For both techniques the posterior wall was sutured followed by suturing of the anterior wall using PDS 5-0. In the CSG two threads were used separately for the anterior and posterior wall, which were tied to each other on both sides of the BEA. The time required to perform the anastomosis was recorded. The surgical technique and perioperative management of patients undergoing BEA have been described in detail^1^. Patients were blinded regarding the random allocation to either CSG or ISG. Similarly, clinicians responsible for the postoperative care and trial personnel were blinded for the suture technique randomization. The operating surgeon could not be masked to the intervention.

### Study population

Patients who were scheduled for an operation with a BEA at the Department of Visceral, Thoracic and Vascular Surgery at University Hospital Dresden between 21 January 2016 and 20 September 2017 were screened for inclusion into the trial. Eligible patients were 18 years or older and met the following inclusion criteria: planned elective BEA, diameter of the CBD of at least 7 mm, and providing written informed consent. Exclusion criteria were previous BEA procedures, impaired mental state, language barrier, lack of compliance, and emergency procedures.

### Objectives and outcomes

The study aimed to compare the benefits and risks of the CS technique and the IS technique for patients undergoing BEA. The primary endpoint of this study was the time required to complete the anastomosis. Secondary outcome parameters were BEA-associated postoperative complications with and without operative revision of the BEA, including bile leakage, cholestasis, and cholangitis. Further secondary outcome parameters were morbidity, including other surgical and non-surgical complications and mortality up to day 30 after the intervention as well as survival. Short-term complications were defined as complications occurring within 1 month after surgery, whereas long-term complications were defined as complications occurring, at the earliest, 1 month up to 24 months after surgery, including anastomotic stenosis, jaundice, and cholestasis. Bile leakage was defined in accordance with the International Study Group of Liver Surgery (ISGLS) definition as elevated drain bilirubin levels, which exceeded at least three times the serum bilirubin concentration or as the need for radiological or operating intervention resulting from biliary collections or biliary peritonitis^20^. Cholestasis was defined as dilatation of the bile ducts diagnosed by ultrasound or CT paired with elevated bilirubin levels or jaundice. Cholangitis was defined as clinical or laboratory signs of infection paired with elevated bilirubin levels or jaundice. Jaundice was classified as a clinical diagnosis. Stenosis of the BEA was defined as clinical symptoms of impaired bile transport paired with radiological evidence of anastomosis stenosis. Follow-ups were performed at 6, 12, and 24 months after surgery. The 6-month follow-up included laboratory values as well as a clinical assessment, including medical history and physical examination regarding long-term complications. At the 12- and 24-month follow-ups a clinical assessment without laboratory values was performed. All data were collected and assessed by trial personnel and study doctors who were blinded to the suture technique.

### Statistical analysis

Statistical analysis was performed using R version 4.0.1 (R Project for Statistical Computing, Vienna, Austria), while GraphPad Prism version 8 (GraphPad Software, La Jolla, CA, USA) was used for graphical illustrations. Data were analysed using the intention-to-treat population. Assuming a difference of 11 min suture time between the two groups and s.d. of 22 min, a total of 38 patients per intervention group were calculated (1:1 ratio) to be necessary for primary analysis with a significance level α = 0.05 and a power of (1 − β) = 0.8 (two-sided test) based on previous studies^18^. To account for possible drop-outs, a total of *n* = 80 (40 per arm) were aimed to be included. Continuous variables are presented as median with interquartile range (i.q.r.). Differences in continuous variables were assessed using a two-tailed Mann–Whitney *U* test. Categorical variables were summarized as absolute and relative frequencies (percentages) and compared using the chi-squared test or exact Fisher’s exact test depending on sample size (Fisher’s test was used if the expected number of cases in one cell was fewer than five). Two-tailed *P* values <0.050 were considered statistically significant. Effect sizes for categorical outcomes are reported by OR with 95 per cent confidence interval (c.i.). Binary logistic regression was used to calculate the OR of continuous data. OR of continuous variables are presented with 95 per cent c.i. and unit of increase (u.o.i.).

## Results

### Patient characteristics

**Fig. 1:**
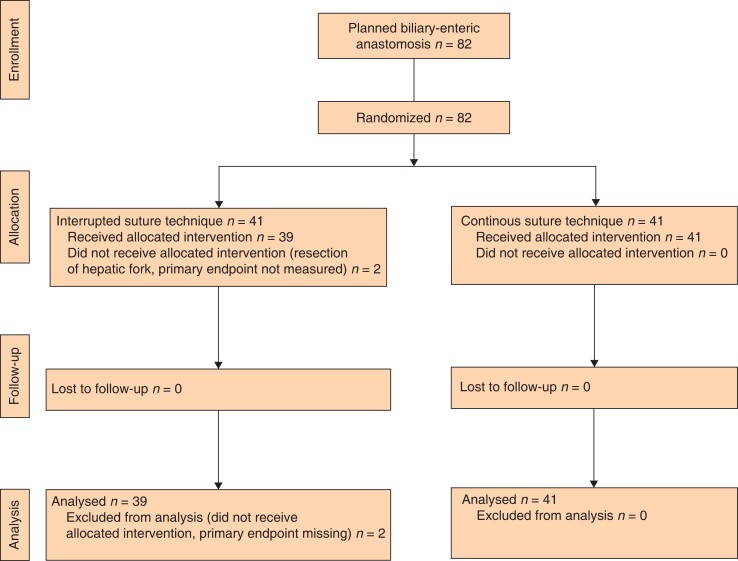
Flow chart of patient enrolment, randomization and follow-up

In total, 82 patients were assessed for eligibility, 41 patients were enrolled and randomized in the CSG and 41 patients in the ISG of which two patients did not receive the allocated intervention (*Fig. 1*). The dates of the first and last patient randomization were 21 January 2016 and 9 September 2017, respectively. Baseline characteristics of all patients, underlying disease, co-morbidities, and preoperative blood test results are summarized in *Table 1*. Baseline physical constitution assessed by the ASA physical status classification system and the Eastern Cooperative Oncology Group (ECOG) scores were comparable in both groups. Surgical indications were balanced in both groups, as well as the pre-existing medical co-morbidities. There were no differences in the preoperative serum levels of bilirubin, alkaline phosphatase (ALP), aspartate-aminotransferase (AST), and alanine aminotransferase (ALT) between the two treatment groups.

**Table 1:** Patient characteristics

	Continuous suture	Interrupted suture	*P*
	(*n* = 41)	(*n* = 39)	
**Age (years), median (i.q.r.)**	72 (65.5–77)	67 (63–76)	0.267
**BMI (kg/m^2^), median (i.q.r.)**	24.7 (22.7–22.9)	24.5 (27.3–29.1)	0.725
**Sex ratio (M:F)**	20:21	17:22	0.809
**ASA class**			0.740
I	3 (7.3)	1 (2.6)	
II	12 (29.3)	11 (28.2)	
III	26 (63.4)	27 (69.2)	
**ECOG scale**			0.235
0	17 (41.5)	10 (25.6)	
1	21 (51.2)	27 (69.3)	
2	3 (7.3)	2 (5.1)	
**Disease**			0.795
Pancreatic carcinoma	20 (48.8)	22 (56.4)	
Extrahepatic bile duct carcinoma	8 (19.5)	9 (23.1)	
Duodenal carcinoma	3 (7.3)	3 (7.7)	
Inflammatory disease	2 (4.9)	2 (5.1)	
Ampulla of Vater carcinoma	7 (17.1)	3 (7.7)	
Recurrent ampulla carcinoma	1 ( 2.4)	0 (0.0)	
**Cardiovascular co-morbidities**	14 (34.1)	14 (35.9)	1.000
**Pulmonary co-morbidities**	5 (29.4)	6 (26.1)	1.000
**Chronic renal failure**	3 (17.6)	7 (30.4)	0.471
**Preoperative interventions**	3 (17.6)	7 (30.4)	0.471
Common bile duct stent	24 (58.5)	20 (51.3)	0.822
ERC	30 (73.2)	25 (64.1)	0.726
PBD	0 (0.00)	1 (2.56)	0.803
**Diabetes mellitus**	10 (58.8)	15 (65.2)	0.934
**Preoperative blood test results**			
Bilirubin level (mg/dl), median (i.q.r.)	15.8 (6.6–44.1)	17.0 (9.7–145.7)	0.267
ALP level (U/l), median (i.q.r.)	2.2 (1.5–6.0)	3.9 (1.7–6.2)	0.725
AST level (U/l), median (i.q.r.)	0.5 (0.4–1.4)	0.7 (0.5–1.3)	0.084
ALT level (U/l), median (i.q.r.)	0.7 (0.4–1.5)	1.0 (0.4–3.1)	0.158

Values are *n* (%) unless otherwise indicated. i.q.r., interquartile range; ECOG, Eastern Cooperative Oncology Group; ERC, endoscopic retrograde cholangiopancreatography; PBD, percutaneous biliary drainage; ALP, alkaline phosphatase; AST, aspartate transaminase; ALT, alanine aminotransferase.

### Operating characteristics and primary outcome

Operating data, including suture time, are outlined in *Table 2*. The surgical procedures were equally distributed between both groups. For biliary reconstruction an omega loop was used in 61 (76.25 per cent) patients and a Roux-en-Y hepaticojejunostomy in 19 (23.75 per cent). Although there was a higher proportion of median laparotomies in the ISG, the difference in surgical incisions was not significant. There was no difference between groups in both duct diameter and distance to the biliary confluence; however, the time required to complete the BEA was significantly longer in the ISG compared with the CSG (median (i.q.r.) difference 10.37 min, 22.4 (15.0–28.0) min *versus* 12.0 (10.0–17.0), *P* < 0.001, OR 1.26; 95 per cent c.i. 1.13 to 1.40). The difference (3.31 min) in the CSG for large (12 mm or larger, 14.48 min (10.99–19.00)) CBD was not different compared with small CBD (less than 12 mm, 11.17 min (9.71–14.75), *P* = 0.083). There was no time difference between large and small CBDs in the ISG (21.21 min (16.08–25.75) *versus* 24.55 min (14.46–29.59), *P* = 0.972).

**Table 2:** Perioperative details

	Continuous suture	Interrupted suture	*P*
	(*n* = 41)	(*n* = 39)	
**Surgical access**			0.054
Transverse	39 (95.2)	33 (84.6)	
Median	1 ( 2.4)	6 ( 15.4)	
L-shaped	1 ( 2.4)	0 ( 0.0)	
**Operation intent**			1.000
Curative	38 ( 92.7)	37 ( 94.9)	
Palliative	3 ( 7.3)	2 ( 5.1)	
**Procedure**			0.733
Whipple procedure	7 (17.1)	7 (17.9)	
PPPD	25 (61.0)	22 (56.4)	
Extended liver resection	1 (2.4)	0 (0.0)	
Palliative biliary-enteric anastomosis	1 (2.4)	2 (5.1)	
Total pancreatectomy	3 (7.3)	1 (2.6)	
Bile duct resection	4 (9.8)	5 (12.8)	
Other	0 (0.0)	2 (5.1)	
**Time for anastomosis (min), median (i.q.r.)**	12.0 (10.0–17.0)	22.4 (15.0–28.0)	<0.001
**Distance to biliary confluence (mm), median (i.q.r.)**	13.5 (10.0–16.8)	15.0 (10.0–18.0)	0.785
**Duct diameter (mm), median (i.q.r.)**	12.0 (10.0–17-8)	14.0 (10.0–18.0)	0.560

Values are *n* (%) unless otherwise indicated. PPPD, pylorus preserving pancreaticoduodenectomy; i.q.r., interquartile range.

### Short-term complications after BEA

Surgical complications independent of the BEA were similarly distributed between the two treatment groups. The most common complications in both groups were bile leakage, pancreatic fistula, and wound infection (*Table 3*). Bile leakage occurred in six patients in the CSG (14.6 per cent) and in four patients in the ISG (10.3 per cent; *P* = 0.738). In the CSG three patients had a grade B bile leakage and three patients had a grade C bile leakage. The three patients with the grade B bile leakage were treated with PTCD and one of these patients also received CT-guided drainage. The patients with grade C bile leakage were reoperated on and received a new BEA. In the ISG one patient had a grade A bile leakage, two patients had a grade B bile leakage, and one patient had a grade C bile leakage. One patient with a grade B bile leakage was treated with PTCD and one patient received CT-guided drainage. The patient with a grade C bile leakage received an operation revision with a new BEA. Cholestasis and cholangitis occurred only once in the CSG (2.4 per cent) and not in the ISG (0 per cent; *P* = 1.000). Three patients in the CSG had liver abscesses (7.3 per cent) and none in the ISG (0 per cent; *P* = 0.241). All biliary complications were distributed among the total of six patients in the CSG (14.6 per cent) and four patients (10.3 per cent) in the ISG. Further complications such as pancreatic fistula, gastrointestinal leakage, and respiratory, cardiac, or renal complications were equally distributed between the groups (*Table 3*). Postoperative blood test results of bilirubin, ALP, AST, ALT, and γ-glutamyltransferase (GGT) on postoperative day 1 and 3 were similar in both groups (*Table 3*). Further, the mortality up to day 30 after the intervention was similarly distributed between the two treatment groups (*Table 3*). One patient in the CSG died due to pneumonia with multi-organ failure on postoperative day 21.

**Table 3:** Short-term complications

	Continuous suture	Interrupted suture	*P*	OR	95% c.i.
	(*n* = 41)	(*n* = 39)			
**Biliary-enteric anastomosis -associated complications**					
Cholestasis	1 (2.4)	0 (0.0)	1.000	0.98	0.93–1.02
Bile leakage	6 (14.6)	4 (10.3)	0.738	0.67	0.17–2.57
Cholangitis	1 (2.4)	0 (0.0)	1.000	0.98	0.93–1.02
Liver abscess	3 (7.3)	0 (0.0)	0.241	0.93	0.85–1.01
**Other surgical complications**					
Biochemical leakage/pancreatic fistula (total)	8 (19.5)	10 (25.6)	0.698	1.42	0.5–4.09
Grade A	2 (4.88)	5 (12.8)			
Grade B	4 (9.76)	4 (10.3)			
Grade C	2 (4.88)	1 (2.6)			
Small intestine anastomosis leakage	1 (2.4)	0 (0.0)	1.000	0.98	0.93–1.02
Gastroenterostomy leakage	1 (2.4)	2 (5.1)	0.611	2.16	0.19–24.85
Abdominal wall abscess	3 (7.3)	3 (7.7)	1.000	1.06	0.2–5.57
Abdominal wall dehiscence	3 (7.3)	2 (5.1)	1.000	0.68	0.11–4.34
**Non-surgical complications**					
Respiratory failure	4 (9.8)	5 (12.8)	0.734	1.36	0.34–5.49
Cardiopulmonary complication	4 (9.8)	2 (5.1)	0.676	0.50	0.09–2.90
Renal complication	2 (4.9)	2 (5.1)	1.000	1.05	0.14–7.87
**30-day mortality**	1 (2.4)	0 (0.0)	1.000	0.98	0.93–1.02
**Blood test results (POD1)**					
Bilirubin level (mg/dl), median (range)	19.3 (10.4–33.5)	19.9 (9.4–83.9)	0.433	1.01	1.00–1.02
ALP level (U/l), median (range)	1.4 (1.–3.1)	1.8 (1.1–3.6)	0.523	1.02	0.88–1.19
AST level (U/l), median (range)	1.4 (1.–2.5)	1.4 (1.2–2.2)	0.725	0.95	0.85–1.07
ALT level (U/l), median (range)	1.2 (0.6–2.3)	1.2 (0.8–2.0)	0.977	0.95	0.81–1.11
GGT level (U/l), median (range)	1.9 (0.7–5.0)	3.4 (0.9–6.8)	0.114	1.02	0.94–1.12
Drainage bilirubin level (mg/dl), median (range)	13.3 (10.2–21.6)	22.4 (10.4–60.6)	0.217	1.01	0.99–1.02
**Blood test results (POD3)**					
Bilirubin level (mg/dl), median (range)	9.3 (5.7–21.5)	12.2 (7.9–36.6)	0.070	1.01	1.00–1.03
ALP level (U/l), median (range)	1.4 (1.0–2.6)	1.6 (1.2–3.1)	0.308	1.05	0.83–1.34
AST level (U/l), median (range)	0.7 (0.6–1.2)	0.8 (0.6–1.2)	0.534	1.16	0.93–1.44
ALT level (U/l), median (range)	0.8 (0.5–1.4)	0.9 (0.6–1.5)	0.638	1.06	0.90–1.25
GGT level (U/l), median (range)	1.1 (0.6–2.4)	2.3 (0.7–5.2)	0.100	1.04	0.91–1.19
Drainage bilirubin level (mg/dl), median (range)	15.4 (11.8–24.6)	14.6 (11.0–49.2)	0.418	1.00	0.99–1.01

Values are *n* (%) unless otherwise indicated. c.i., confidence interval; POD, postoperative day; ALP, alkaline phosphatase; AST, aspartate transaminase; ALT, alanine aminotransferase; GGT, γ-glutamyl transferase.

### Long-term complications after BEA

The median follow-up time of all patients in the study group who did not drop out due to death, was 24.37 months (i.q.r. 24.31–24.49, range 6.1–25.23). The median overall survival was 22.67 (i.q.r. 10.26–24.37, range 0.7–25.23). During follow-up, 19 (46.3 per cent) patients died in the CSG, and 19 (48.7 per cent) in the ISG. In the CSG, 21 (51.2 per cent) patients, of those that did not drop out due to death, finished the follow-up and 20 (51.3 per cent) patients in the ISG. There was no significant difference in median overall survival time between the CSG (21.33 months (i.q.r. 10.03–24.37), range 0.7–24.73) and the ISG (22.80 months (i.q.r. 11.62–24.37), range 1.3–25.23). Within 24 months of follow-up, no anastomotic stenosis occurred in the CSG or ISG (*Table 4*). Cholestasis was recorded in two patients of the CSG (4.9 per cent) and three patients of the ISG (7.7 per cent; *P* = 0.940). Jaundice was diagnosed in one patient of the CSG (2.4 per cent) and in four patients of the ISG (10.3 per cent; *P* = 0.409). None of the patients in both groups developed cholestasis or jaundice as a result of BEA stenosis but because of tumour recurrence or liver metastasis. One patient in the ISG developed jaundice because of liver failure without cholestasis. Overall, two patients (4.9 per cent) in the CSG and four patients (10.3 per cent) in the ISG developed long-term complications; however, no significant difference for any long-term complications between the two groups was found.

**Table 4:** Long-term complications

	Continuous suture	Interrupted suture	*P*	OR	95% c.i.
	(*n* = 41)	(*n* = 39)			
**Blood test results (6 months after surgery)**					
Bilirubin level (mg/dl), median (range)	7.0 (5.9–12.9)	8.9 (5.8–12.2)	0.687	1.01	1.0–1.03
ALP level (U/l), median (range)	2.0 (1.7–3.6)	2.1 (1.5–4.2)	0.838	1.05	0.83–1.34
AST level (U/l), median (range)	0.5 (0.4–0.9)	0.5 (0.4–0.7)	0.455	1.16	0.93–1.44
ALT level (U/l), median (range)	0.4 (0.3–0.8)	0.5 (0.4–0.8)	0.383	1.06	0.9–1.25
GGT level (U/l), median (range)	0.7 (0.5–1.9)	1.4 (0.7–4.4)	0.099	1.04	0.91–1.19
**24 months after surgery**					
Biliary-enteric anastomosis stenosis	0 (0.0)	0 (0.0)	1.000		
Cholestasis	2 (4.9)	3 (7.7)	0.940	1.63	0.26–10.29
Jaundice	1 (2.4)	4 (10.3)	0.409	4.57	0.49–42.85

Values are *n* (%) unless otherwise indicated. c.i., confidence interval; ALP, alkaline phosphatase; AST, aspartate transaminase; ALT, alanine aminotransferase; GGT, γ-glutamyl transferase.

## Discussion

In this prospective, randomized clinical study, a significantly shorter suture time for the continuous compared with the IS technique was found; however, no significant difference in short-term postoperative complications between both groups was observed. Bile leakage, the most common short-term biliary complication after BEA that can necessitate interventional or surgical treatment, occurred in six (14.6 per cent) patients of the CSG and four (10.3 per cent) patients in the ISG. Furthermore, only one patient in the CSG (2.4 per cent) and no patient in the ISG developed a cholangitis as a short-term complication. Three patients in the CSG developed liver abscesses, but none in the ISG. Looking at the long-term complications, including jaundice, cholestasis, and BEA stenosis, there was no difference between the two groups. During follow-up, 19 (46.3 per cent) patients in the CSG and 19 (48.7 per cent) in the ISG died due to their oncological disease; the shorter survival is a possible confounder masking differences in such long-term complications. Furthermore, the distance of the BEA to the biliary confluence was not associated with short-term or long-term complications in both groups. A possible reason might be that only the CHD and not the CBD was used for the BEA. The blood supply of the BEA was always good and a risk factor for stenosis was eliminated. Therefore, the length of the remaining hepatic duct has not been confirmed as a risk factor for anastomotic leakage in this study.

These findings are in line with a retrospective study that compared continuous and IS techniques for choledochocholedochostomy during liver transplantation in 100 patients and reported an incidence of biliary complications of 16.1 per cent for the CSG and 13.2 per cent for the ISG, with no significant difference between the two groups^12^. Another recent non-randomized study also showed no differences between the techniques regarding short-term and long-term outcomes, but a slightly increased cost in the ISG in patients receiving pancreaticoduodenectomy or total pancreatectomy^18^. Other previous retrospective studies have reported a bile leakage frequency of 3–10 per cent^17,21,22^. The high variance between the studies is partly due to the lack of a standardized definition, which is why in the present study bile leakage was graded according to the ISGLS. Further, a recent systematic review and meta-analysis on cholangitis after BEA reported a pooled rate of postoperative cholangitis of 10 per cent, which is notably higher than in this study^10^. Biliary stenosis of the BEA is a rare long-term complication, and no patient in this study developed biliary stenosis. In a retrospective analysis, biliary stenosis was reported in 2.6 per cent of 1595 patients who underwent pancreaticoduodenectomy^2^, while another study reported a 7.4 per cent rate in patients undergoing pancreaticoduodenectomy for benign indications^23^. Another prospective study comparing different suture techniques with a follow-up protocol of more than 2 years observed anastomotic strictures in 7.4 per cent of patients with CS and in 8.6 per cent of patients with the IS technique. The anastomotic stricture occurred after a median time of 13.5 months^18^.

A limitation of the present study is that only common hepatic ducts with a diameter of at least 7 mm were included. Furthermore, the limited patient number limits the power of secondary outcomes. Further studies, including small and fragile bile ducts where biliary stenosis is more common are needed to determine the complication rates of CS *versus* IS in smaller BEA. Furthermore, biliary stenosis often occurs only years after surgery, which is why further long-term observation studies after CS or IS are necessary to determine the long-term outcomes; however, based on the results of the study at hand and the current knowledge, CS of BEA is equally safe but faster compared with IS.

## Data Availability

The authors confirm that the data supporting the results in the paper will be accessible upon request from the corresponding author.
